# Midkine as a novel prognostic and therapeutic target in meningioma: insights from single-cell analysis and organoid-based drug validation

**DOI:** 10.1186/s12967-026-08068-3

**Published:** 2026-04-14

**Authors:** Pei-Ran Li, Fei-Yang Chen, Lai-Rong Song, Jun-Ting Zhang, Zhen Wu, Wei Chen, Liang Wang

**Affiliations:** 1 Department of Neurosurgery, Beijing Tiantan Hospital, Capital Medical University, Beijing, China; 2 China National Clinical Research Center for Neurological Diseases, Beijing, China; 3 Key Laboratory of Biomechanics and Mechanobiology , (Beihang University) Ministry of Education, Beijing, China; 4 Key Laboratory of Innovation and Transformation of Advanced Medical Devices, Ministry of Industry and Information Technology, Beijing, China; 5 National Medical Innovation Platform for Industry-Education Integration in Advanced Medical Devices (Interdiscipline of Medicine and Engineering), Beijing, China; 6 School of Biological Science and Medical Engineering, Beihang University, Beijing, China; 7 Department of Neurosurgery, Ningxia Hui Autonomous Region People’s Hospital, Ningxia, China

**Keywords:** Meningioma, Midkine, Organoid, Recurrence

## Abstract

**Background:**

Recurrent meningiomas lack effective treatments beyond surgery and radiotherapy, necessitating novel prognostic biomarkers and therapeutic targets.

**Methods:**

Single-cell RNA sequencing coupled with the Scissor algorithm, along with bulk transcriptomic analysis, was used to identify recurrence-associated genes in meningiomas. Clinical prognostic value was assessed using WB, ELISA, and IHC. Functional validation involved MDK knockdown and pharmacological inhibition with iMDK in IOMM-Lee and CH157 meningioma cells. Multiplex immunofluorescence staining was used to verify immune cell infiltration. Meningioma organoids were generated to evaluate the efficacy of iMDK.

**Results:**

MDK, a key gene of recurrence-associated subclusters, is significantly overexpressed in recurrent meningiomas. Plasma MDK levels are markedly elevated in patients with recurrence propensity. IHC confirmed higher MDK expression in recurrent cases, which, after adjusting for confounding factors, correlates with shorter progression-free survival. Knockdown and iMDK administration reduced proliferation and clonogenicity in IOMM-Lee and CH157 meningioma cells. Additionally, high-MDK meningiomas exhibited immunosuppressive features, including reduced CD8^+^/CD4^+^ T-cell infiltration. Furthermore, iMDK induced structural disintegration of meningioma organoids and cell death.

**Conclusions:**

MDK is a key recurrence marker and participates in meningioma progression, promoting proliferation and immunosuppression. Targeting MDK effectively inhibits tumor growth and induces organoid disintegration, highlighting its therapeutic potential.

**Supplementary Information:**

The online version contains supplementary material available at 10.1186/s12967-026-08068-3.

## Background

Meningiomas represent the most common primary tumors of the central nervous system, accounting for approximately 37% of all primary intracranial tumors. Although approximately 75–80% of meningiomas are benign (World Health Organization (WHO) grade 1), the remaining 20–25% exhibit invasive growth or recurrence (WHO grades 2/3) [1–6]. Patients with benign meningiomas generally have a favorable prognosis after surgical resection; however, some patients still exhibit recurrence characteristics. In contrast, patients with high-grade meningiomas have a high recurrence rate even after surgical resection and postoperative radiotherapy. Statistics show that the 10-year progression-free survival rate is 75%-90% for WHO grade 1 meningiomas, 23%-78% for grade 2 meningiomas, and 0% for grade 3 meningiomas [7]. According to the 4th edition of the WHO Classification of Tumors of the Central Nervous System, the overall recurrence rate after resection of benign meningiomas is 7%-25%, while that of atypical and anaplastic meningiomas is 29%-52% and 50%-94%, respectively [8]. Recent clinical studies have explored the potential of molecularly targeted agents (e.g., everolimus, alpelisib, trametinib, capivasertib, vismodegib, bevacizumab) and immunotherapies (e.g., pembrolizumab, nivolumab) [9–12]. Despite these efforts, no agents have achieved ESMO Scale for Clinical Actionability of molecular Targets (ESCAT) I recommendation. Only the mTOR inhibitor everolimus is classified as an ESCAT IIB recommendation [10]. Therefore, identifying new therapeutic targets hold significant clinical importance.

Research on the immune microenvironment of meningiomas has increased in recent years, revealing the diversity of tumor-infiltrating immune cells and their roles in tumor progression [13–17]. The meningioma immune landscape comprises various immune cell types, including CD3 + T cells, CD4 + T helper cells, CD8 + cytotoxic T lymphocytes, and CD163 + macrophages [18, 19]. Among these, T cells represent the predominant lymphocyte population. Compared with WHO grade 1 meningiomas, grade 3 tumors exhibit a significant reduction in the density of CD3+, CD4+, and CD8 + T lymphocytes [20]. The density of tumor-infiltrating lymphocytes (TILs) is closely associated with tumor grade and patient prognosis. Studies indicate that higher densities of CD3 + and CD8 + TILs correlate with improved recurrence-free survival [16].

Organoids, as three-dimensional in vitro models, can recapitulate the tissue architecture, cellular heterogeneity, and microenvironmental characteristics of tumors in vivo, providing a vital tool for cancer research [21, 22]. In the field of drug efficacy evaluation, organoid models demonstrate superior predictive accuracy for in vivo drug response and toxicity compared to traditional 2D models. This capability accelerates the translation of potential therapeutic agents from preclinical research to clinical application [23–28].

MDK, a secreted heparin-binding growth factor and pleiotropic cytokine, is implicated in diverse pathophysiological processes including inflammation, tissue repair, and cancer [29]. In malignancies, elevated MDK expression not only promotes tumor cell proliferation and migration but also modulates the immune microenvironment to facilitate cancer progression. Furthermore, MDK is closely associated with tumor recurrence and metastasis and may serve as a non-invasive biomarker for progression-free survival. In central nervous system tumors, MDK promotes glioblastoma recurrence by sustaining the self-renewal and tumorigenic capacity of glioma-initiating cells [30]. Additionally, MDK participates in the neuro-immuno-cancer axis of low-grade gliomas, where it activates CD8 + T cells to paradoxically foster tumor growth [31]. In non-small cell lung cancer (NSCLC), MDK demonstrates a strong association with hypoxia-inducible factor 1-alpha (HIF-1α), driving tumor angiogenesis and metastasis [32]. Colorectal cancer (CRC) patients exhibit significantly elevated serum MDK levels correlating with poorer survival rates. Studies suggest MDK’s utility as a biomarker for both CRC screening and prognostic assessment [33]. Similarly, MDK overexpression in bladder cancer underscores its potential as a non-invasive diagnostic tool, particularly for patients presenting with microscopic hematuria [34]. Nevertheless, the specific expression profile of MDK in meningiomas—especially recurrent cases—and its potential as a therapeutic target or prognostic indicator remain elusive. Elucidating these aspects constitutes the primary objective of the present study.

## Methods

### Patient samples and data collection

Surgical resection tissue specimens and matched clinicopathological data were retrospectively collected from 90 pathologically confirmed meningioma patients, with a total of 119 specimens, at Beijing Tiantan Hospital, Capital Medical University, between January 2016 and June 2025. Notably, multiple specimens derived from the same patients were allocated to different experimental procedures. The distribution of specimens utilized for specific assays was as follows: Five-year recurrence/non-recurrence comparison: 21 specimens; Enzyme-Linked Immunosorbent Assay (ELISA): 20 specimens; Immunohistochemistry (IHC): 48 specimens; Multiplex immunofluorescence staining: 24 specimens; Meningioma Organoids: 6 specimens. Internal dataset single-cell RNA sequencing data were derived from our previous study [35]. External validation datasets including single-cell RNA sequencing and bulk transcriptome datasets were obtained from the Gene Expression Omnibus (GEO) under accession numbers GSE183655 and GSE136661, respectively. Detailed analytical pipelines for both single-cell RNA sequencing and bulk transcriptomic data are described in our prior publications [35–37]. Written informed consent was obtained from all participating patients. This study was approved by the Institutional Review Board of Beijing Tiantan Hospital. Clinical information of all patients is shown in Tables S1-S5.

### Candidate gene identification

Scissor algorithm was used to screen the cell subpopulations most strongly associated with the recurrent phenotype (alpha = 0.05, family = “cox”), followed by identification of their differential genes [38].

### BayesPrism

The BayesPrism algorithm was employed for deconvolution of different cellular states [39]. Based on the characteristics of distinct cellular states identified at the single-cell level, the cellular content of different clusters in the GSE136661 dataset was evaluated.

### ProjecTIL

The ProjecTIL algorithm was used to analyze the functional status of T cells in meningioma single-cell transcriptome data [40]. By integrating patients’ single-cell RNA sequencing data with a predefined T cell reference atlas, differences in the distribution of T cell subsets among patients with different recurrence statuses were compared.

### Preference of scissor1 and scissor2 in immune cell composition

We quantified the preference of Scissor1 and Scissor2 in immune cell composition by calculating the ratio of observed to expected cell numbers (Ro/e) for each [41, 42]. Specifically, the expected cell numbers of Scissor1 and Scissor2 in each cluster were derived from the Chi-square test; if the Ro/e of Scissor1 or Scissor2 in a cluster is greater than 1, it indicates that Scissor1 or Scissor2 has a preference for that cluster in terms of immune cell composition.

### PSM

Propensity score matching (PSM) was used to adjust for potential baseline confounding factors. Using the nearest-neighbor method without replacement for PSM, pairs of patients were matched with a caliper of 0.02, std.caliper = F and a ratio of 1:1.

### Cell culture

The human meningioma cell line, IOMM-Lee, was used as the published study [43–45]. CH157 cells were generously donated by Professor Wenbin Li from Beijing Tiantan Hospital, Capital Medical University [46]. IOMM-Lee and CH157 cells were cultured in Dulbecco’s modified Eagle medium (Gibco [Life Technologies, Thermo Fisher Scientific, Rockford, Maryland, USA) with 10% fetal bovine serum (FBS; Gibco [Life Technologies, Thermo Fisher Scientific]) at 37 °C in a humidified environment with 5% carbon dioxide atmosphere. All cell lines were authenticated using short tandem repeat (STR) analysis and were regularly tested for mycoplasma contamination to ensure they were free from contamination (Fig. S1).

### Lentiviral transfection

Cells were seeded in 6-well plates at a density of 5 × 10⁵ cells per well. After 24 h, when the cells reached 70%-80% confluence, they were transfected with lentivirus containing MDK shRNA. After 18 h of incubation at 37 °C, the cells were passaged and seeded into new 6-well plates. Thirty-six hours after cell passage, the cells were selected using puromycin (1 µg/mL, A1113803; Gibco). The MDK shRNA sequence is 5′- CGACTGCAAGTACAAGTTTGA − 3′.

### RT-qPCR

Total RNA was extracted from cells using TRIzol reagent (15596018; Invitrogen) after washing with PBS. cDNA was synthesized from purified RNA using a SuperScript III First-Strand cDNA synthesis system (18080051; Invitrogen) according to the manufacturer’s instructions. SYBR Green PCR Master Mix (Q331-02; Vazyme) was used for PCR amplification and a real-time PCR instrument (iQ5, Bio-Rad Laboratories) was used to quantify the expression of mRNA. GAPDH served as an endogenous control, and mRNA expression levels were quantified using the 2-ΔΔCt method. All primer sequences are shown in Table S6.

### WB

Proteins were extracted using SDS cell lysis buffer (P0013G; Beyotime) supplemented with a protease inhibitor cocktail (87785; Thermo Scientific). Protein quantification was measured using the Pierce BCA Protein Assay Kit (PC0020; Solarbio). Next, equal amounts of proteins were loaded and subjected to electrophoresis. Following blocking, the membrane was incubated with primary antibody overnight at 4 °C. Subsequently, secondary antibody was incubated for 60 min at room temperature. The protein bands were detected using the ECL Western Blotting Substrate (Solarbio). Detailed procedures are described in our previous study [36]. Information on the antibodies used is provided in Table S7. The full uncropped blots images can be found in Fig. S2.

### ELISA

Plasma MDK levels in meningioma patients were quantified using a commercial ELISA kit (EK1253; MULTISCIENCES) according to the manufacturer’s protocol. Venous blood (5 mL) was collected preoperatively into EDTA-coated tubes. Plasma was isolated by centrifugation at 3,000 × *g* for 15 min at 4 °C. Plasma samples (diluted 1:5) and standards were loaded in triplicate into antibody-precoated 96-well plates. After washing unbound materials, biotinylated detection antibody was added and incubated. Following removal of unbound antibody, streptavidin-conjugated horseradish peroxidase (Streptavidin-HRP) was added. After washing, 3,3’,5,5’-tetramethylbenzidine substrate was added for chromogenic reaction (protected from light). The reaction was stopped with stop solution. Absorbance was measured at 450 nm with reference wavelength correction (570–630 nm). MDK concentrations (ng/mL) were calculated by extrapolation from the standard curve.

### Half-maximal inhibitory concentration (IC₅₀) assay

The half-maximal inhibitory concentration (IC₅₀) of the MDK inhibitor iMDK (HY-110171; MedChemExpress) was determined via Cell Counting Kit-8 (CCK-8) assay. Briefly, cells were seeded in 96-well plates at 2 × 10³ cells/well and cultured for 24 h. Subsequently, cells were treated with iMDK at twelve concentration gradients (0.1–100 µM) for 48 h, with six replicate wells per concentration along with blank (cell-free) and negative (drug-free) controls. After incubation, 10 µL CCK-8 reagent was added to each well followed by 1-hour incubation at 37 °C. Absorbance was then measured at 450 nm using a microplate reader. For analysis, cell viability (%) was calculated and dose-response curves were fitted using GraphPad Prism to derive IC₅₀ values.

### EdU assay

Cellular proliferation was assessed in primary meningioma cells using the EdU assay (C0078S; Beyotime). Briefly, cells were seeded in 6-well plates and cultured until reaching 60%–70% confluency. Subsequently, the medium was replaced with EdU-containing medium (50 µM) for a 2-hour incubation. Following treatment, cells were fixed with 4% paraformaldehyde at room temperature for 20 min, then permeabilized with 0.3% Triton X-100 for 15 min. For fluorescence labeling, the Click-iT EdU detection kit was employed: reaction buffer containing Alexa Fluor 594-conjugated azide was added according to manufacturer’s instructions, followed by a 30-minute incubation in the dark. After washing, nuclei were counterstained with Hoechst 33,342 (1 µg/mL) for 10 min. Finally, five random fields per well were imaged at 200× magnification using fluorescence microscopy, and the percentage of EdU-positive cells was quantified using ImageJ software.

### Colony formation

IOMM-Lee and CH157 cells (1000 cells/well) were seeded in a 6-well plate. After the cells had adhered, they were treated with different concentrations of the drug, and the medium was changed every 3 to 4 days thereafter. Colonies were stained with crystal violet after10 days.

### Establishment, culture, and drug administration of meningioma organoids

The establishment of meningioma organoids (MOs) was performed with reference to the method described by Yamazaki and Fadi Jacob et al. [28, 47]. Surgically resected meningioma tissues were placed in sterile normal saline at 4 °C, promptly transferred to the laboratory, and processing was initiated within 1 h. The tissues were rinsed with PBS to remove blood contaminants, and necrotic tissues were excised. The tissues were cut into 0.5–1 mm pieces using fine scissors, and the pieces were collected and rinsed. After treatment with red blood cell lysis buffer and subsequent rinsing, the tissue pieces were transferred to 6-well plates containing MOs medium, with 10–20 pieces per well. The plates were incubated on a shaker at 120 rpm at 37 °C with 5% CO₂, and approximately 50% of the medium was replaced every 48 h. After approximately 1–2 weeks of culture, round, cell-dense MOs were formed. MOs with good morphology were selected and transferred to 48-well plates. An appropriate amount of MOs medium was added to each well, along with 20 µmol of iMDK, and culturing was continued under the aforementioned conditions for 10 days. After the culture period, the MOs were harvested and embedded. The preparation of MOs medium is as follows: For a 50 ml system, add 23.5 ml of DMEM: F12 medium (Gibco), 23.5 ml of Neurobasal medium (Gibco), 1 ml of B-27 supplement (50×), minus vitamin A (Gibco), 0.5 ml of N2 supplement (100×) (Gibco), 0.5 ml of GlutaMAX (100×) (Gibco), 0.5 ml of NEAA (100×) (Gibco), 0.5 ml of penicillin-streptomycin (100×) (Gibco), 12.5 µl of Human insulin solution (10 mg/ml) (Sigma-Aldrich), and 50 µl of β-Mercaptoethanol (1000×) (Gibco).

### Calcein/PI dual staining assay

Organoid viability was assessed using the Calcein/propidium iodide (PI) dual staining assay (C1367S; Beyotime). Briefly, meningioma organoids with intact morphology were transferred to 48-well plates (200 µL medium/well) and maintained with orbital shaking. Subsequently, staining solution was added to the culture medium at a 1:1000 dilution ratio followed by 30-minute incubation at 37 °C in the dark. After incubation, nuclei were counterstained with PI, and samples were washed three times with PBS. Immediately thereafter, fluorescence microscopy was performed to capture images. For quantification, live cells (green; Calcein AM-positive) and dead cells (red; PI-positive) were enumerated using ImageJ.

### Staining of tissue sections

Paraffin-embedded meningioma tissue and MOs specimens were cut into 5 μm-thick sections for immunohistochemical detection. The procedures were as follows: first, the tissue sections were dewaxed with xylene and gradient ethanol, followed by antigen retrieval using citrate/EDTA buffer; after washing, the sections were blocked with 0.3% hydrogen peroxide and 5% normal goat serum in sequence, and then incubated with primary antibodies at 4 °C overnight; subsequently, the sections were incubated with secondary antibodies at room temperature for 20 min, and then counterstained with hematoxylin solution; finally, the sections were scanned and imaged using Akoya PhenoImager, and ImageJ was used for statistical analysis.

### Organoid viability assays

To evaluate the proliferation-inhibitory effect of iMDK on MOs, the CellTiter-Glo^®^ 3D Cell Viability Assay (Promega, WI, USA) was used. According to the manufacturer’s instructions, MOs were incubated with the assay reagent for 30 min, followed by measurement of luminescence.

### Multiplex immunofluorescence staining

The specific procedures were as described previously [35]. Briefly, using the Absin 5-Color IHC Kit (abs50013; Absin), 5 μm-thick paraffin-embedded meningioma sections were dewaxed in xylene (twice, 15 min each time), hydrated in gradient ethanol (10 min each time), and washed with TBST for 5 min. Antigen retrieval was performed with citrate/EDTA buffer in a microwave for 15 min, and after cooling to room temperature, the sections were washed twice with TBST. Primary antibodies were added and incubated at room temperature for 60 min, followed by three washes with TBST; secondary antibodies were then added and incubated at room temperature for 15 min, with three subsequent washes with TBST; Fluorescent dye staining was conducted for 10 min, followed by three washes with TBST. After re-antigen retrieval, the above steps were repeated. Finally, nuclei were stained with DAPI for 5 min, followed by washing with TBST and ultrapure water for 5 min each, and the sections were mounted and kept away from light. Finally, the stained slices were imaged by scanning with the Axio Scan.Z1.

### Statistical analysis

Statistical analyses were performed using R software and GraphPad. Differences between groups were analyzed using Student’s t-test, Mann-Whitney test, and Wilcoxon test. The Kaplan-Meier method was used to evaluate the survival risk and progression risk in different expression groups. Propensity score matching was performed using the R package MatchIt. Multivariable Cox regression analysis was performed using the R package survival to identify independent risk factors associated with meningioma recurrence. The model incorporated the following variables: age, sex, WHO grade, extent of resection, radiotherapy status, and MDK expression level. Quantitative data were presented as mean ± standard deviation, and categorical data were expressed as percentages. A p-value < 0.05 was considered statistically significant (*p < 0.05;**p < 0.01༛***p < 0.001).

## Results

### MDK as a recurrence biomarker with non-invasive prognostic potential in meningiomas

**Fig. 1:**
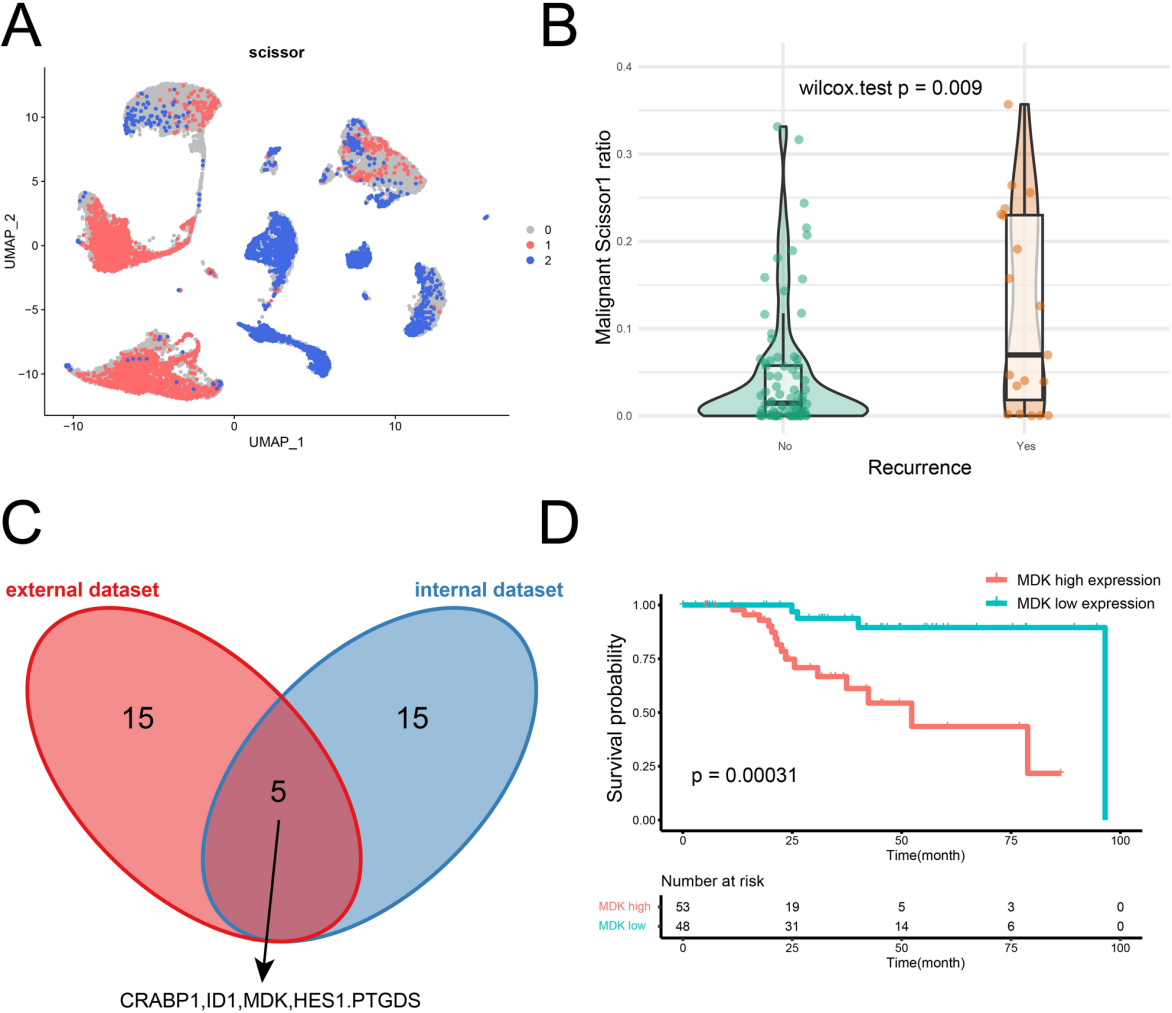
Screening of signature genes in recurrence-associated subclusters. (**A**) UMAP plot showing the distribution of cell subclusters identified by the Scissor algorithm, based on the internal dataset (*n* = 5). (**B**) Violin plot comparing the proportion of Scissor1 between patients with tumor recurrence (Yes) and those without (No), based on deconvolution of the internal dataset. (**C**) Venn diagram displaying the intersection of recurrence-associated genes in the internal and external datasets, with 5 overlapping genes (CRABP1, ID1, MDK, HES1, PTGDS) screened. (**D**) Kaplan-Meier survival curves stratified by MDK expression level; high MDK expression is associated with poor patient prognosis

**Fig. 2:**
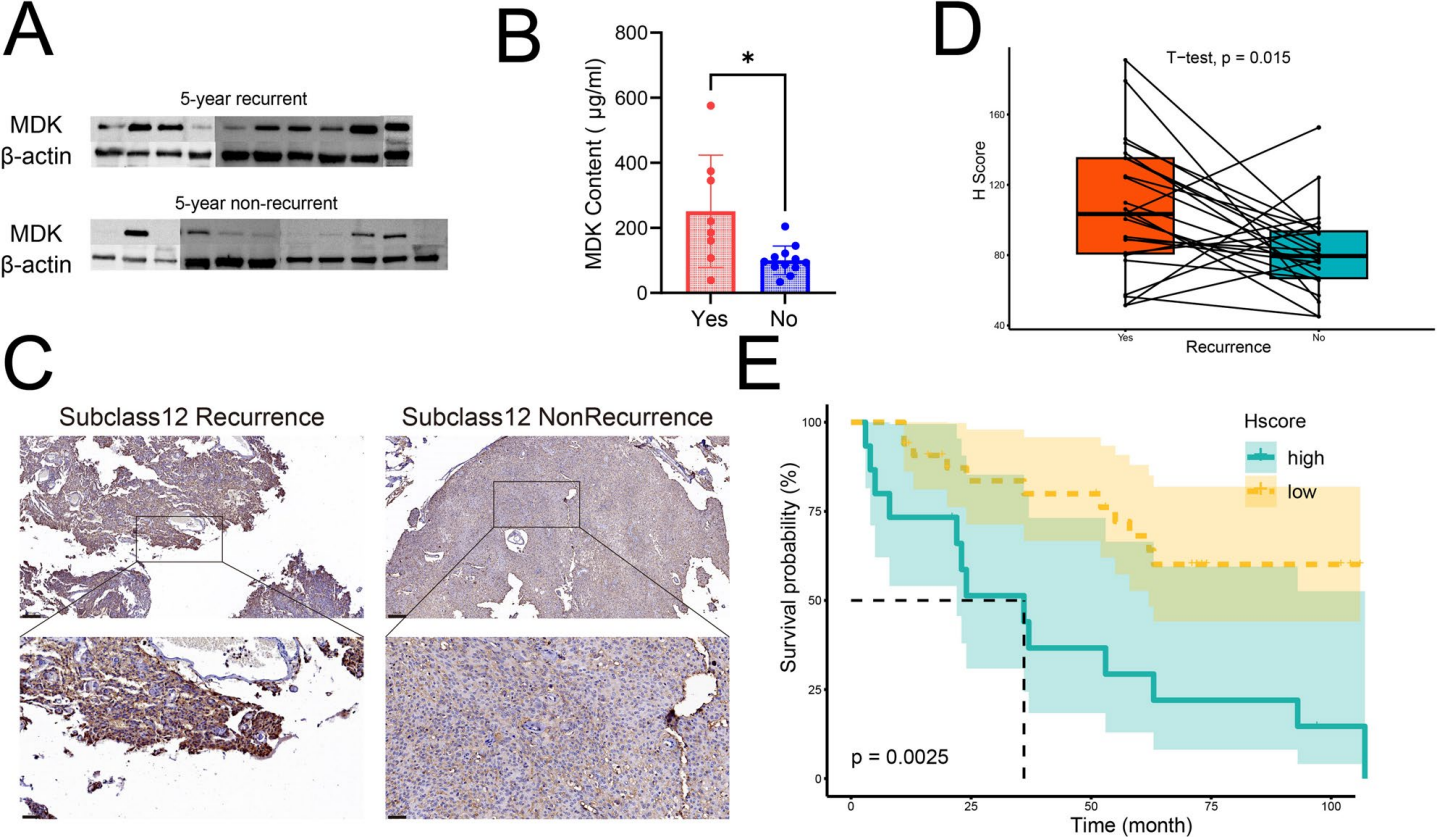
High MDK expression is significantly associated with meningioma recurrence. (**A**) Western blot analysis showing MDK protein expression in tumor tissues with recurrence within 5 years (upper n=10) and without recurrence (lower n=11). (**B**) Box plot demonstrating significantly elevated plasma MDK levels in patients with recurrence propensity (n=8:12). (**C**) Immunohistochemical staining images showing characteristic MDK expression in recurrent and non-recurrent patients. (**D**) Paired box plots comparing the H-scores of MDK in tumors from recurrent and non-recurrent patients (n=24:24). (**E**) Kaplan-Meier survival curves stratified by MDK expression level; high MDK expression is associated with poor patient prognosis (P<0.001)

MDK emerged as a significant recurrence factor in meningiomas through integrated multi-omics analyses and clinical validation. Initially, Scissor algorithm applied to internal and external validation datasets identified recurrence-correlated Scissor1 tumor subpopulations (Fig. 1A, Fig. S3). For specific protocols, Using our internal single-cell RNA sequencing data and a public transcriptomic dataset (GSE136661), we first applied the Scissor algorithm to identify cell subpopulations significantly associated with recurrence (Scissor 1cells). The FindAllMarkers function was then used to identify the top 20 differentially expressed genes within this Scissor 1 subpopulation. To validate this finding, we repeated the identical analytical pipeline—Scissor algorithm followed by differential gene analysis—on an independent external single-cell dataset (GSE183655), again obtaining a list of top 20 marker genes from its Scissor 1 population. MDK expression was markedly elevated in these Scissor1 cells (Table S8, Table S9). Critically, BayesPrism deconvolution confirmed higher proportions of Scissor1 cells in recurrent samples (Fig. 1B, Fig. S4). The intersection of the top 20 recurrence-related differentially expressed genes from both internal and external datasets was obtained, resulting in a total of 5 genes, including CRABP1, ID1, MDK, HES1, and PTGDS (Fig. 1C). Among them, high expression of MDK showed the most significant association with shorter progression-free survival (Fig. 1D, Fig. S5); therefore, MDK was selected as the final candidate gene for further validation. Further validation demonstrated: (i) upregulated MDK protein in recurrent Meningioma via WB (Fig. 2A); (ii) Elevated plasma MDK concentrations were observed in patients with adverse histopathological features via ELISA, which include unclear relationship with brain tissue, adhesion to brain tissue, local pseudopapillary formation around blood vessels, prominent nucleoli, local clear/cystic cell changes, local involvement of striated muscle tissue, and involvement of nerve fiber bundles, suggesting that these patients may have a higher tendency to recur (Fig. 2B). (iii) After adjusting for confounding factors including gender, age, and WHO grade using PSM, the intensity and extent of MDK staining were higher in patients with recurrence via IHC (Fig. 2C and D, Fi. S6). Most notably, MDK-high patients exhibited significantly reduced progression-free survival (Fig. 2E). Multivariate Cox regression analysis confirmed that high MDK expression remains an independent risk factor for shortened progression-free survival (PFS) after adjusting for the aforementioned covariates (HR = 8.35, 95% CI: 2.30–30.21, *p* < 0.001) (Fig. S7). Collectively, these data establish MDK as a biomarker linked to recurrence, with potential as a non-invasive prognostic marker.

### Knockdown and pharmacological inhibition of MDK significantly attenuated IOMM-Lee and CH157 cells proliferation

**Fig. 3:**
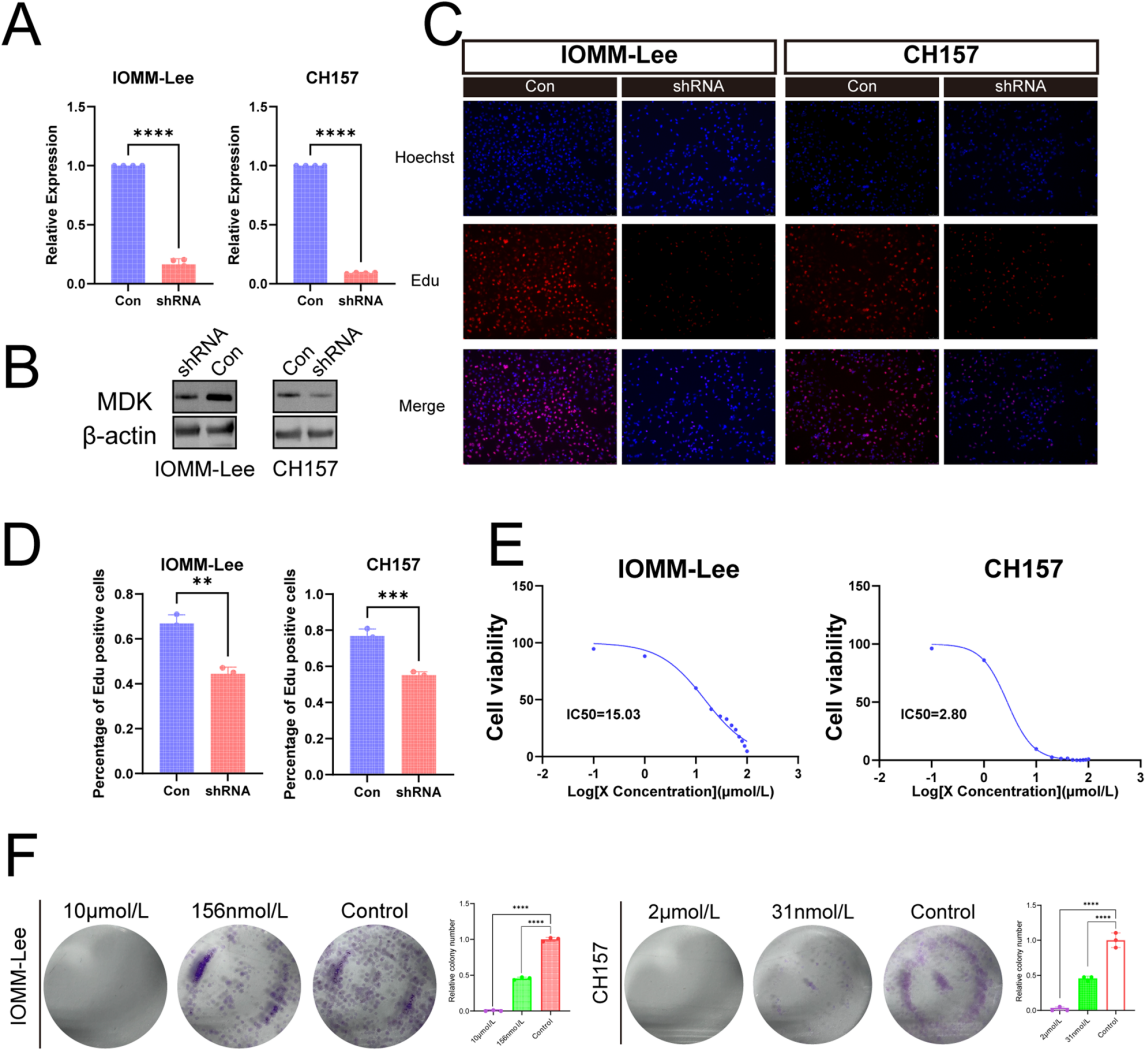
MDK knockdown and iMDK-mediated functional inhibition in IOMM-Lee cells. (**A**) RT-qPCR detecting the relative expression of MDK mRNA in the Con and shRNA group in the IOMM-Lee and CH157 cells. (**B**) Western blot verifying MDK protein levels in the Con and shRNA group in the IOMM-Lee and CH157 cells. (**C**) Representative EdU images of the Con and shRNA group. in the IOMM-Lee and CH157 cells. (**D**) Statistical analysis of the proportion of EdU-positive cells. in the IOMM-Lee and CH157 cells. (**E**) Dose-response curve of iMDK in the IOMM-Lee and CH157 cells. (**F**) Effects of different concentrations of iMDK treatment on colony formation in IOMM-Lee and CH157 cells

Specifically, MDK knockdown significantly reduced both mRNA and protein levels (Fig. 3A and B). Consistently, EdU assays revealed a marked reduction in the proportion of EdU-positive cells in LOMM-Lee and CH157 cells following MDK knockdown (Fig. 3C and D). Consistently, dose-response assays with the MDK inhibitor iMDK revealed IC₅₀ values of 15.03 µM (IOMM-Lee cells) and 2.80 µM (CH157 cells) after 48-hour exposure (Fig. 3E). Results of the colony formation assay showed that, across the 3 tested drug concentration gradients, the colony-forming ability of IOMM-Lee and CH157 cells exhibited a decreasing trend with increasing drug concentration. Moreover, significant inhibition of colony formation was observed even at lower drug concentrations (Fig. 3F). Collectively, both knockdown and pharmacological inhibition of MDK impaired IOMM-Lee and CH157 cells proliferation.

### Elevated MDK expression correlated with diminished CD8⁺ and CD4⁺ T-cell infiltration in meningiomas.

**Fig. 4:**
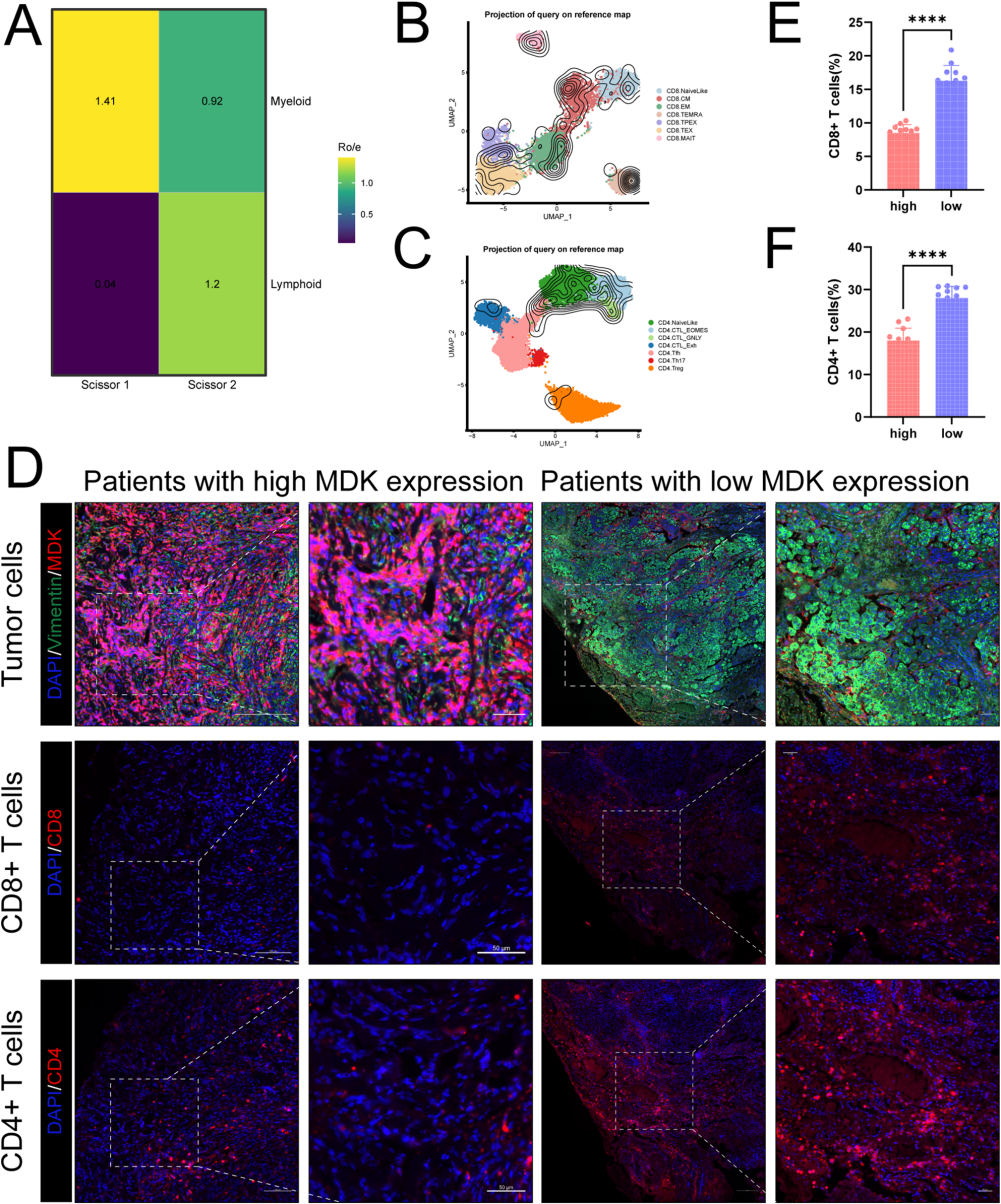
High MDK expression reduces CD8⁺ and CD4⁺ T cell infiltration. (**A**) Ro/e plot comparing the proportional differences of myeloid and lymphoid cells between Scissor 1 and Scissor 2 subtypes, based on the internal dataset. (**B**) Projection analysis of the single-cell dataset onto the CD8⁺ T cell reference atlas, based on the internal dataset. (**C**) Projection analysis of the single-cell dataset onto the CD4⁺ T cell reference atlas, based on the internal dataset. (**D**) Multiplex immunofluorescence staining: upper row shows tumor cells; middle row shows CD8⁺ T cells; lower row shows CD4⁺ T cells, comparing staining results between patients with high MDK expression (left two columns) and low MDK expression (right two columns). (**E**) Statistical analysis of CD8⁺ T cell proportions in groups with high and low MDK expression(*n* = 12:12). (**F**) Statistical analysis of CD4⁺ T cell proportions in groups with high and low MDK expression(*n* = 12:12)

Ro/e analysis revealed no significant difference in the number of myeloid immune cells between Scissor1 and Scissor2, whereas lymphoid immune cells were significantly decreased in Scissor1 (Fig. 4A, Fig. S8). Given the roles of CD8⁺ and CD4⁺ T cells in solid tumors, we used ProjecTIL to explore changes in the composition of CD8⁺ and CD4⁺ T cell subsets between Scissor1 and Scissor2 (Fig. 4B and C, Fig. S9). Compared with Scissor2, both CD8⁺ and CD4⁺ T cells were significantly reduced in Scissor1, suggesting the presence of immune suppression-related factors in Scissor1 (Table S10- Table S13). MDK may play a role in this process. To verify the relationship between MDK expression and immune cell infiltration, multiplex immunofluorescence staining was performed (Fig. 4D). Critically, this immunosuppressive landscape was spatially validated, demonstrating markedly reduced infiltration of both CD8⁺ T cells and CD4⁺ T cells within MDK-enriched regions (Fig. 4E and F). Collectively High MDK expression is spatially associated with reduced infiltration of CD8⁺ and CD4⁺ T cells.

### iMDK induces MOs disintegration and cell death

**Fig. 5:**
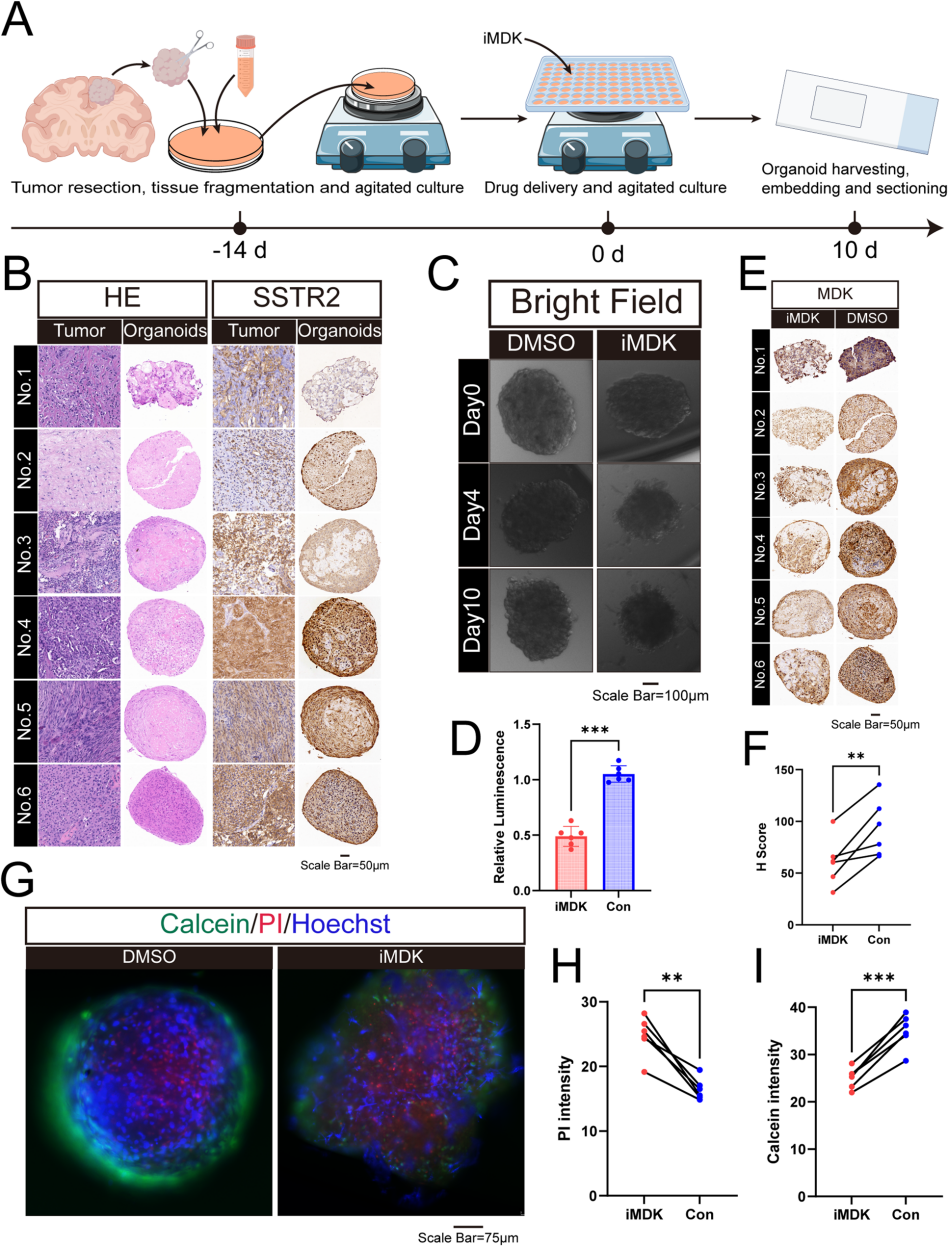
iMDK promotes disintegration and death of MOs. (**A**) Schematic diagram of MOs construction, culture, and drug treatment, created by Figdraw. (**B**) HE (left) and SSTR2 (right) staining in parental tumor tissues and MOs (*n* = 6). (**C**) Bright-field images showing the growth status of MOs treated with DMSO (left column) and iMDK (right column) on days 0, 4, and 10. (**D**) CellTiter-Glo 3D Cell Viability Assay demonstrating iMDK proliferation inhibitory effect on MOs (*n* = 6). (**E**) MDK expression in MOs after treatment with iMDK (left column) and DMSO (right column) (*n* = 6). (**F**) H-scores of MDK immunohistochemistry in MOs treated with iMDK and Con group (*n* = 6). (**G**) Representative images of Calcein/PI dual staining in iMDK-treated and Con MOs. (**H**) Statistical analysis of Calcein fluorescence intensity (*n* = 6). (**I**) Statistical analysis of PI fluorescence intensity (*n* = 6)

The establishment process of MOs is illustrated in Fig. 5A. Representative images of meningioma organoids are provided in Fig. S10. The imaging features of all patients are provided in Fig. S11. HE and SSTR2 staining confirmed retention of parental tumor architectural patterns (Fig. 5B, Fig. S12). Following 10-day iMDK exposure (20 µM), striking morphological alterations were observed: MOs displayed structural disintegration, peripheral cell shedding, and irregular contours (Fig. 5C, Fig. S13). The CellTiter-Glo 3D Cell Viability Assay further confirmed the proliferation-inhibitory effect of iMDK on MOs (Fig. 5D). Meanwhile, the intensity and extent of MDK staining were significantly reduced (Fig. 5E and F). Furthermore, Calcein/PI dual staining was used to detect live and dead cells in MOs (Fig. 5G). Compared with the control group, iMDK administration resulted in a significant decrease in calcein intensity and an increase in PI staining intensity, indicating its role in promoting cell death (Fig. 5H and I).

## Discussion

This study, by integrating single-cell transcriptomic analysis with functional organoid models, provides the first preliminary evidence highlighting the central role of MDK in meningioma recurrence and demonstrates its dual potential as both a prognostic biomarker and a therapeutic target. We not only present statistical evidence establishing MDK as an independent prognostic factor but also conceptually validate the feasibility of targeting MDK using the pharmacological tool iMDK, thereby providing a rationale for developing novel therapeutic avenues.

The prognostic value of MDK is underscored by its significant overexpression in recurrent tumors, which correlates with poorer survival; this association persists after PSM, highlighting its utility in recurrence risk stratification. Notably, elevated plasma MDK levels in patients with adverse histopathological features further support its potential as a non-invasive liquid biopsy marker. This finding carries particular translational weight: while MDK has documented prognostic roles in CRC and bladder cancers, these malignancies already have established biomarkers in clinical use (CEA/CA19-9 for CRC; BTA/NMP22 for bladder cancer) [33, 34, 48, 49]. In stark contrast, meningiomas lack reliable non-invasive prognostic tools. Plasma MDK quantification via routine ELISA presents a clinically actionable, cost-effective solution for dynamic recurrence risk assessment, offering significant advantages for real-world implementation.

MDK participates in meningioma progression through dual oncogenic mechanisms: intrinsic proliferative promotion and extrinsic immune suppression. Direct pro-tumorigenic effects were evidenced by significantly inhibited proliferation in MDK-knockdown and iMDK administration in IOMM-Lee and CH157 cells, with IC₅₀ values (15.03 µM for IOMM-Lee and 2.80 µM for CH157 cells) supporting its druggability. Notably, while iMDK exhibits broad anti-tumor activity across malignancies, its mechanisms diverge: In NSCLC, it suppresses metastasis and extends survival [32]; In multiple carcinomas, it reverses IFN-γ-induced EMT and metastasis [50]; In ovarian cancer, it synergizes with IFN-γ to inhibit growth and EMT-driven dissemination [51]; In multiple myeloma, it induces dose-dependent cell cycle arrest and apoptosis [52]; In HCC, it not only reduces tumor growth and M2 macrophage polarization but also potentiates lenvatinib efficacy while mitigating toxicity [53].

Single-cell transcriptome analysis and multiplex immunofluorescence confirmed spatial exclusion of T cells within MDK-enriched regions, suggesting MDK-mediated immune evasion as a recurrence factor. Divergent immunomodulatory roles of MDK in other cancers further contextualize our findings: CD151-MDK regulates macrophage accumulation in inflammatory breast cancer [54], glioblastoma MDK networks drive immunosuppressive macrophage polarization [55], lung adenocarcinoma employs MDK-NCL signaling for aneuploidy-associated immune crosstalk [56], and HCC links MDK to Treg/NK imbalance [57]. Collectively, our work uncovers MDK as a unique dual-function orchestrator in meningiomas, simultaneously fueling intrinsic growth and extrinsic immune evasion—a mechanistic paradigm distinct from its roles in other solid tumors.

MOs validated the therapeutic potential of MDK targeting, demonstrating both efficacy and translational promise. Treatment with iMDK induced significant structural disintegration of MOs, reduction in Calcein positive viable cells and increase in PI positive dead cells. Mechanistically, iMDK, as a PI3K inhibitor, suppresses MDK expression [53, 58, 59]. Critically, given the established role of PI3K-AKT-mTOR signaling in meningioma pathogenesis and treatment resistance [60–65], our findings position iMDK as a compelling candidate for combination therapy with mTOR inhibitors such as everolimus. Such combinatorial strategies may offer synergistic efficacy against high-grade, recurrent, or histologically aggressive meningiomas, bridging preclinical validation to clinical application.

For meningiomas with multiple recurrences, as the number of recurrences increases, the tumor’s adhesion to and invasion of surrounding blood vessels, nerves, and brain tissue become more severe. In such cases, surgical resection may not necessarily benefit the patient and is often no longer the first-line treatment. Moreover, most patients at this stage have a history of radiotherapy, and repeated radiotherapy is also limited in application. Drug therapy may be the only option. This study establishes MDK as a pivotal marker of meningioma recurrence, with its dual pro-tumorigenic and immunosuppressive mechanisms providing a rational basis for novel combination therapies. Nevertheless, several limitations warrant consideration. Firstly, the sample size—particularly for single-cell RNA sequencing (*n* = 9) and patient-derived organoids (*n* = 6)—may constrain generalizability. Secondly, mechanistic insights remain incomplete; downstream effectors mediating MDK’s control over proliferation and immune evasion require further validation. Thirdly, the absence of in vivo models precludes assessment of iMDK’s systemic efficacy and safety profiles. Collectively, addressing these limitations will accelerate the translation of MDK-targeted strategies into clinical trials for recurrent meningiomas.

## Conclusions

First, MDK is a key molecular marker for the recurrence of meningiomas, and its high expression is significantly associated with poor prognosis. Second, MDK participates in meningioma progression by promoting tumor cell proliferation and shaping an immunosuppressive microenvironment, as evidenced by the reduced infiltration of CD8⁺/CD4⁺ T cells. Third, targeting MDK has shown potential in inhibiting tumor growth and promoting organoid disintegration in in vitro cell experiments and organoid models, highlighting its translational medical value.

## Supplementary Information

Below is the link to the electronic supplementary material.

Supplementary Material 1

Supplementary Material 2

## Data Availability

The datasets used and/or analysed during the current study are available from the corresponding author on reasonable request.

## Abbreviations

MDK: Midkine
WHO: World Health Organization
ESCAT: ESMO Scale for Clinical Actionability of molecular Targets
NSCLC: Non-small cell lung cancer
CRC: Colorectal cancer
GEO: Gene Expression Omnibus
PSM: Propensity score matching
IC₅₀: Half-maximal inhibitory concentration
MOs: Meningioma organoids
PI: Propidium iodide
