# Supplementary material for: Midkine as a novel prognostic and therapeutic target in meningioma: insights from single-cell analysis and organoid-based drug validation
Source: J Transl Med. 2026 Apr 14;24:535. doi: 10.1186/s12967-026-08068-3 (PMC13088525; doi:10.1186/s12967-026-08068-3)
Supplement: Supplementary file 1 — Supplementary Material 1 [file 12967_2026_8068_MOESM1_ESM.docx]

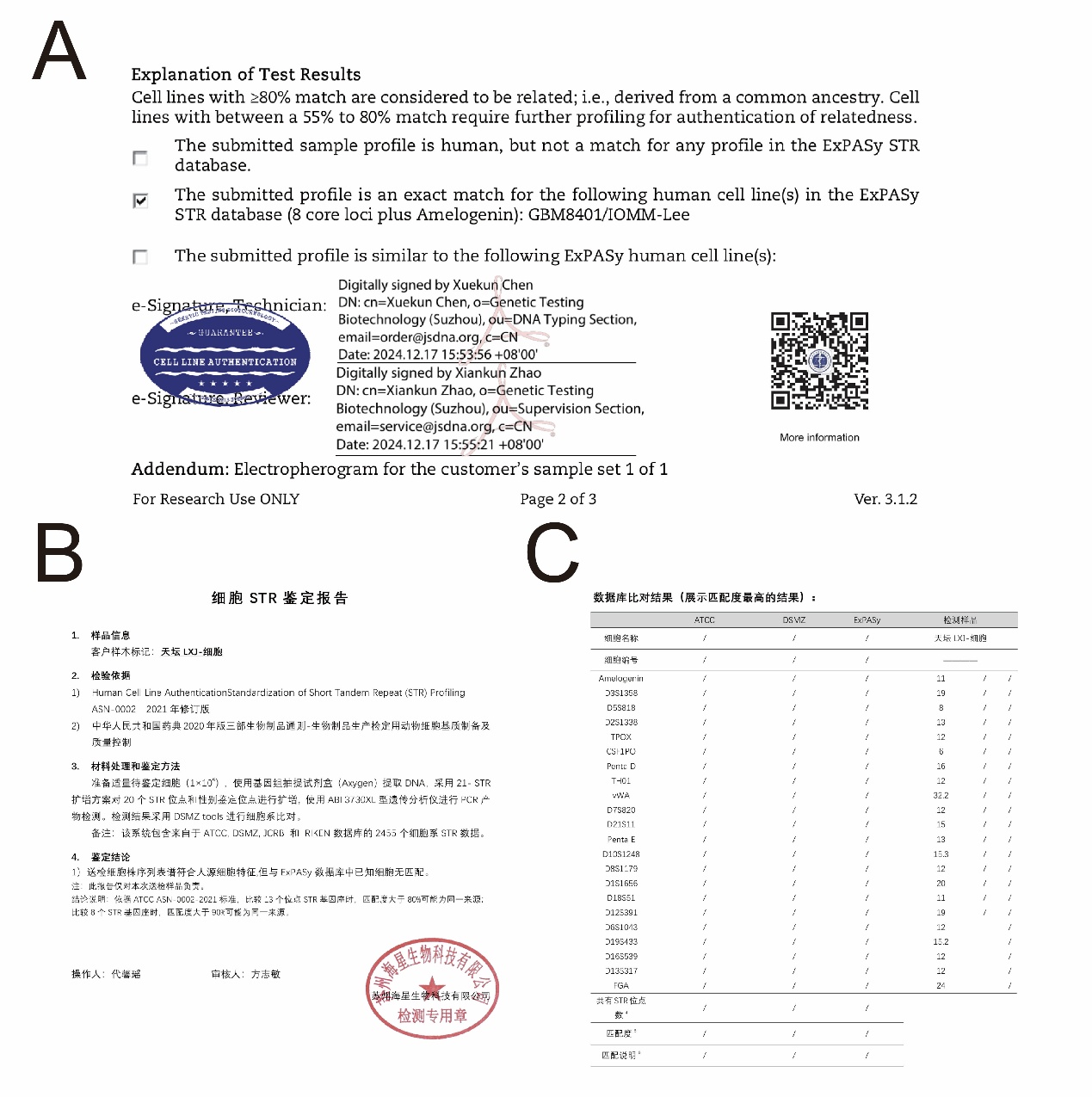


Additional file 1: Figure S1. the STR profiling results for both the IOMM-Lee and the CH157 cell lines

A. STR Profiling Results of the IOMM-Lee Cell Line

B. STR Profiling Results of the CH157 Cell Line, the STR profile confirms its human origin; however, it does not match any known cell line in the reference databases (e.g., ExPASy)

C. Database comparison results of CH157 cell line


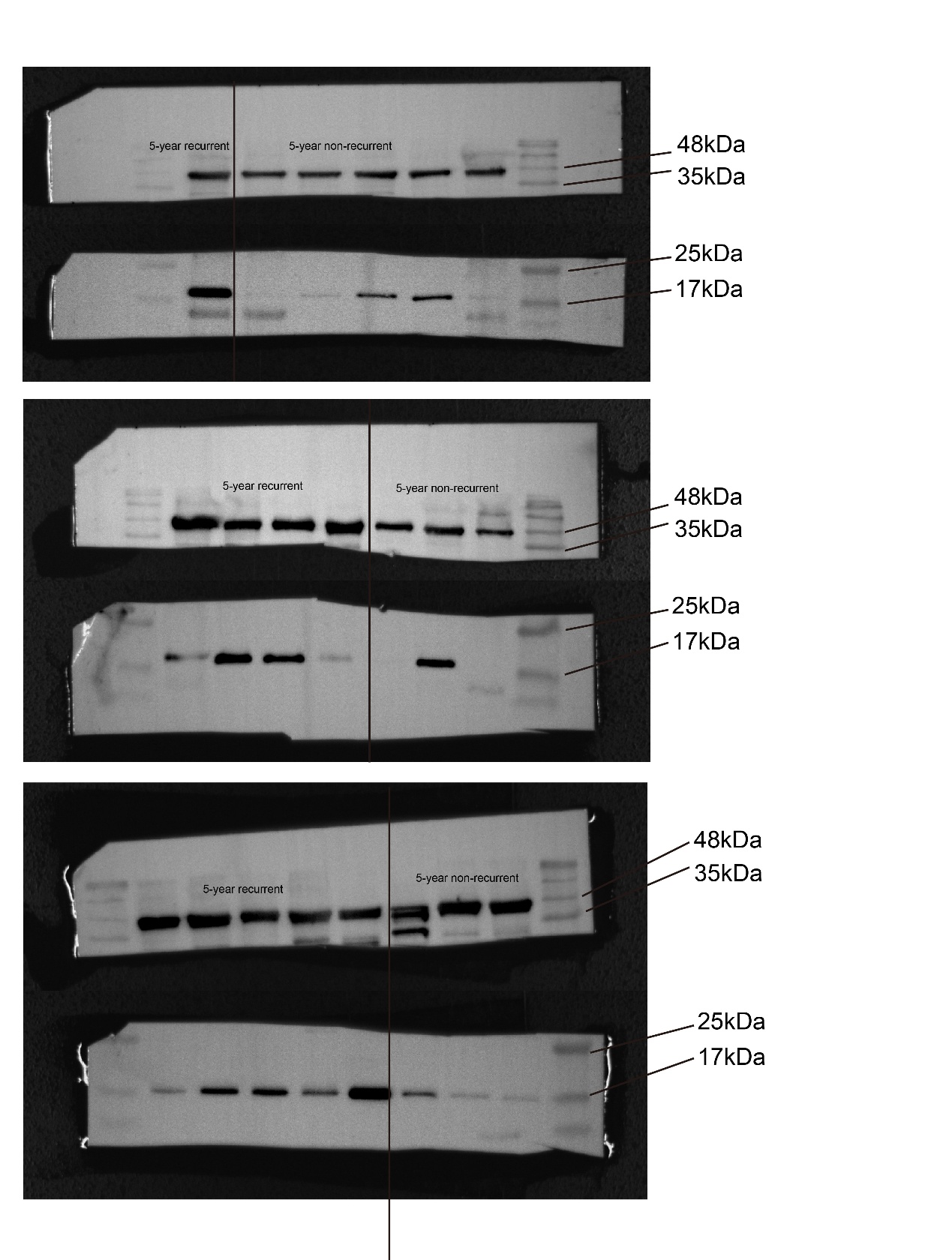


Additional file 2: Figure S2. The full uncropped blots images


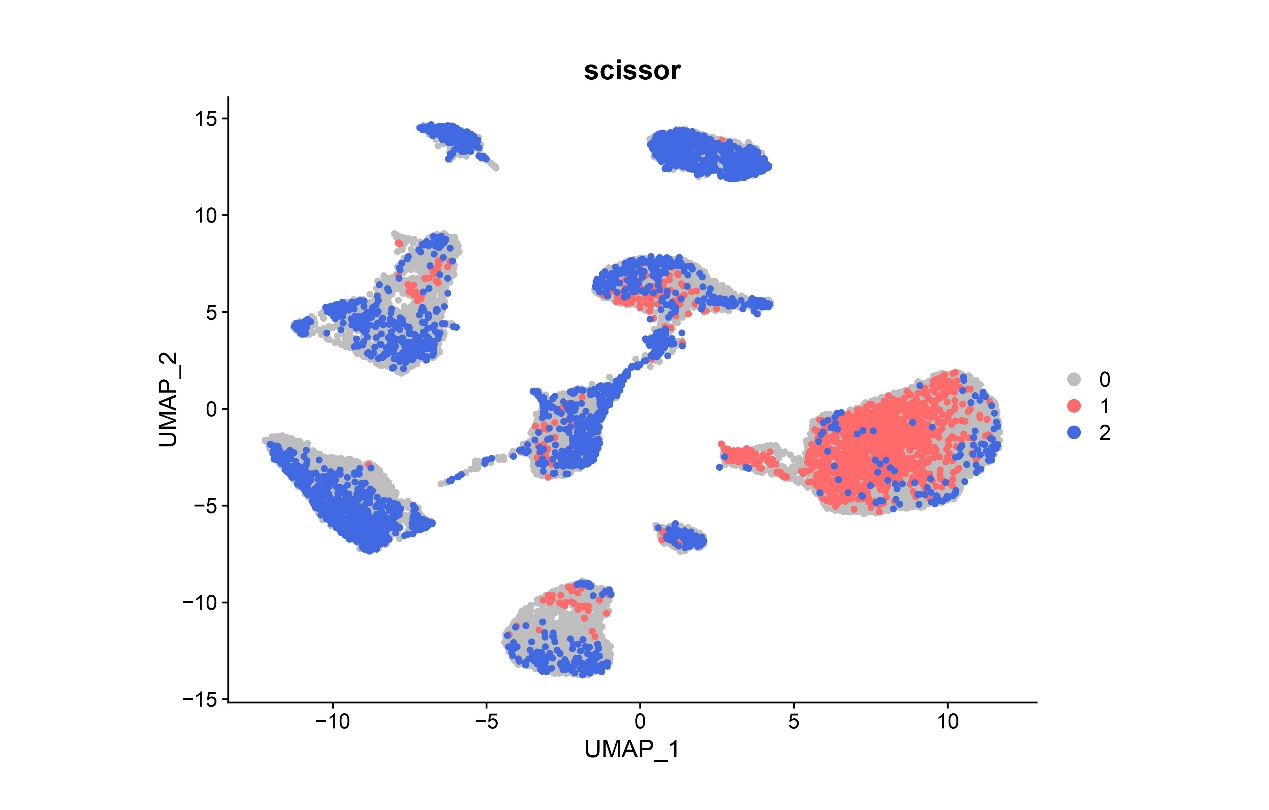


Additional file 3: Figure S3. UMAP plot showing the distribution of cell subclusters identified by the Scissor algorithm, based on the external dataset (n=4).


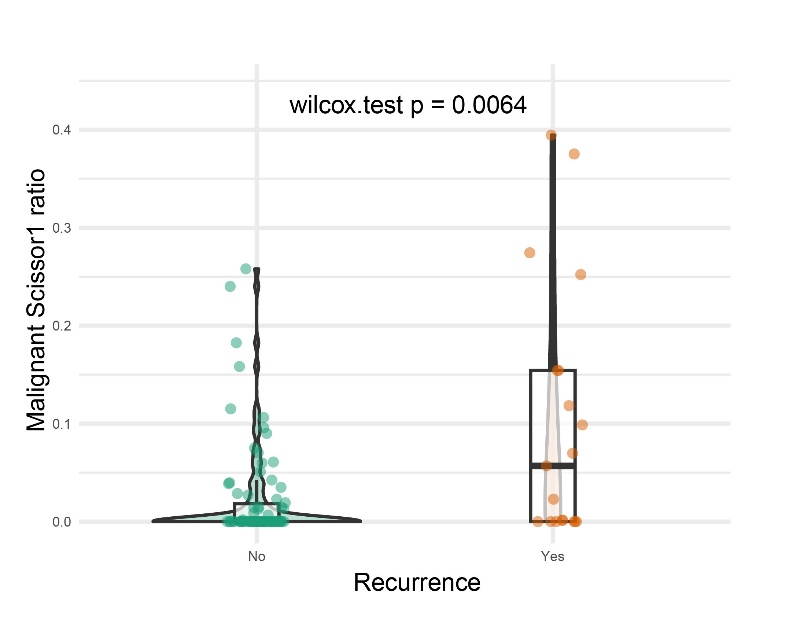


Additional file 4: Figure S4. Violin plot comparing the proportion of Scissor1 between patients with tumor recurrence (Yes) and those without (No), based on deconvolution of the external dataset.


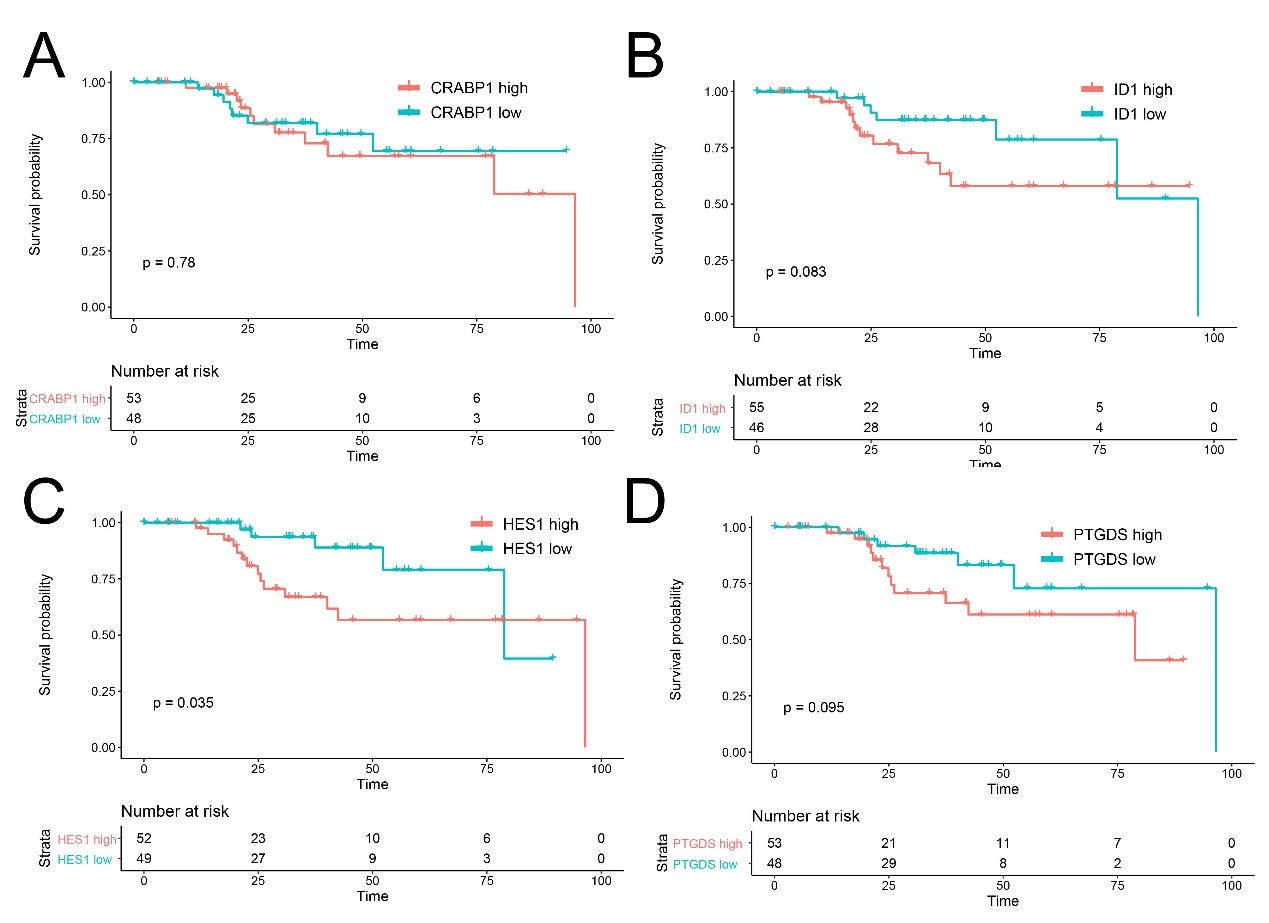


Additional file 5: Figure S5. Association between overlapping gene expression and survival prognosis
A. Kaplan-Meier survival curves for groups with high and low CRABP1 expression.

B. Kaplan-Meier survival curves for groups with high and low ID1 expression.

C. Kaplan-Meier survival curves for groups with high and low HES1 expression.

D. Kaplan-Meier survival curves for groups with high and low PTGDS expression.


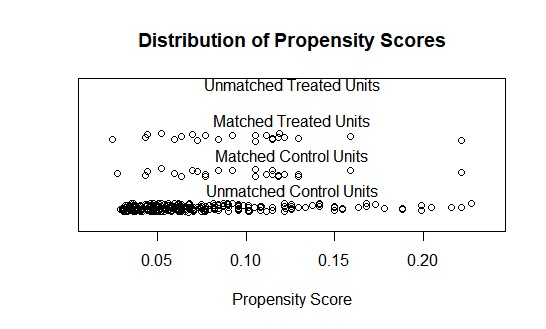


Additional file 6: Figure S6. Scatter plot showing the distribution of propensity scores, distinguishing the score distributions of unmatched treated units, matched treated units, matched control units, and unmatched control units.


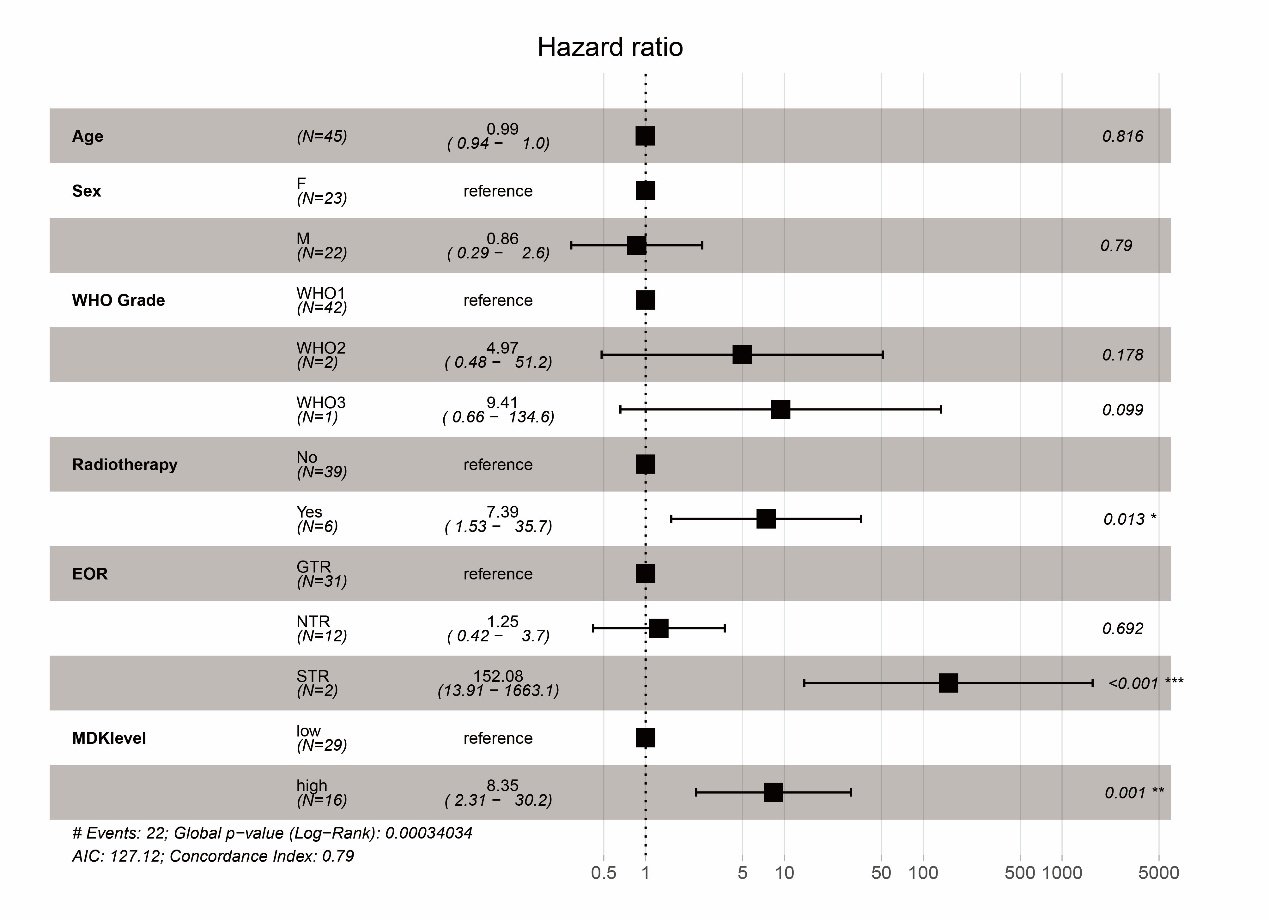


Additional file 7: Figure S7. Multivariable Cox regression analysis was performed in the PSM cohort (n=48). After excluding 3 patients due to missing radiotherapy status data, the final analysis included 45 patients.


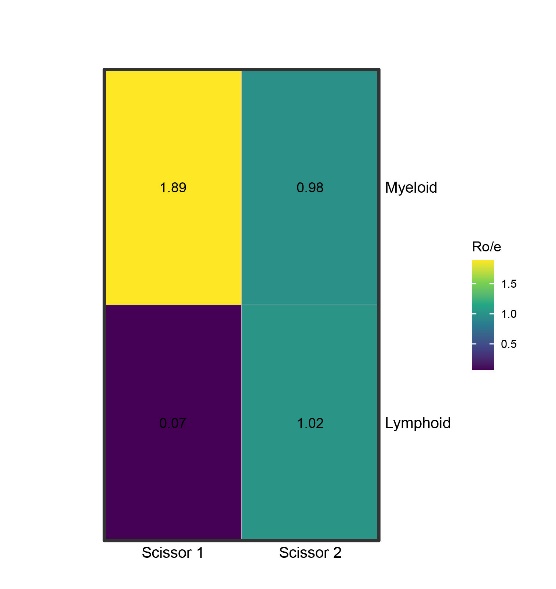


Additional file 8: Figure S8. Ro/e plot comparing the proportional differences of myeloid and lymphoid cells between Scissor 1 and Scissor 2 subtypes, based on the external dataset.


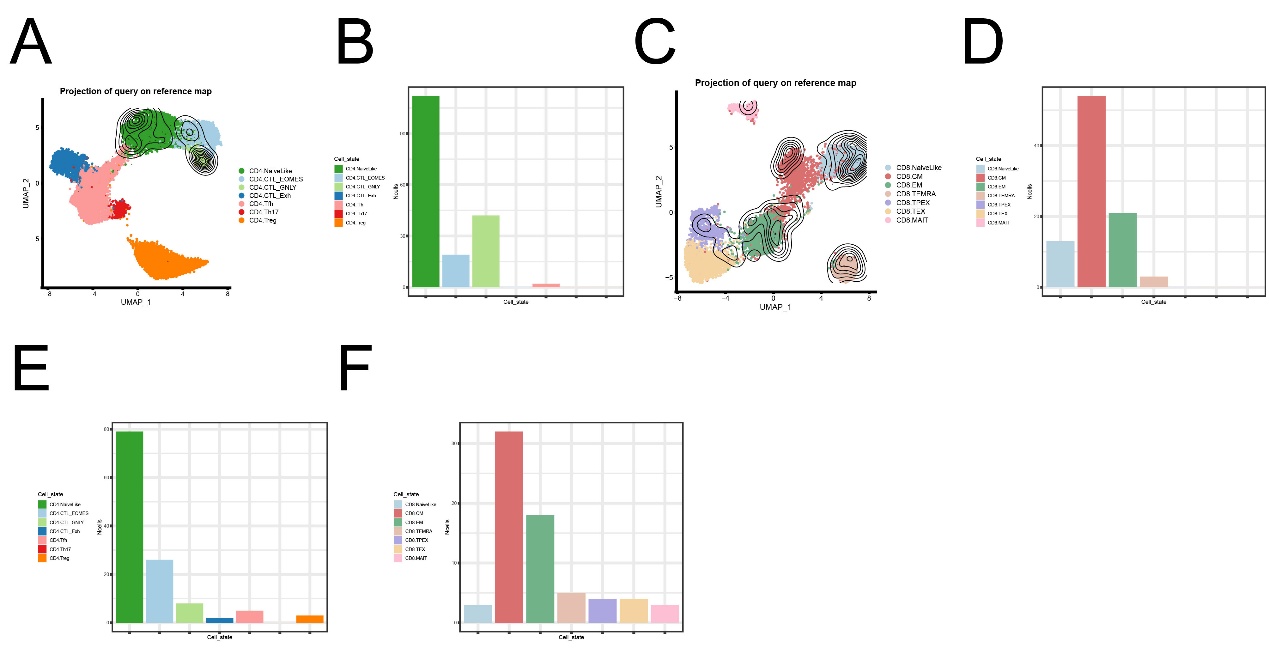


Additional file 9: Figure S9. Projection of single-cell T cell subsets onto reference atlases
A. Projection analysis of the single-cell dataset onto the CD4⁺ T cell reference atlas, based on the external dataset.
B. Bar chart showing the number of each state of CD4⁺ T cells, based on the external dataset.
C. Projection analysis of the single-cell dataset onto the CD8⁺ T cell reference atlas, based on the external dataset.
D. Bar chart showing the number of each state of CD8⁺ T cells, based on the external dataset.

E. Bar chart showing the number of each state of CD4⁺ T cells, based on the internal dataset.

F. Bar chart showing the number of each state of CD8⁺ T cells, based on the internal dataset.


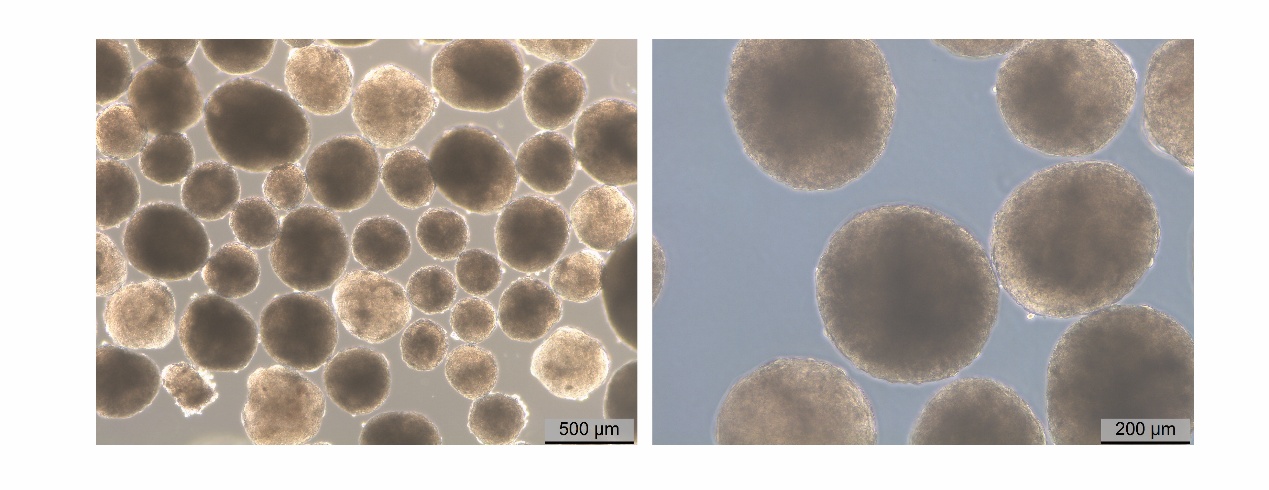


Additional file 10: Figure S10. Representative Images of MOs


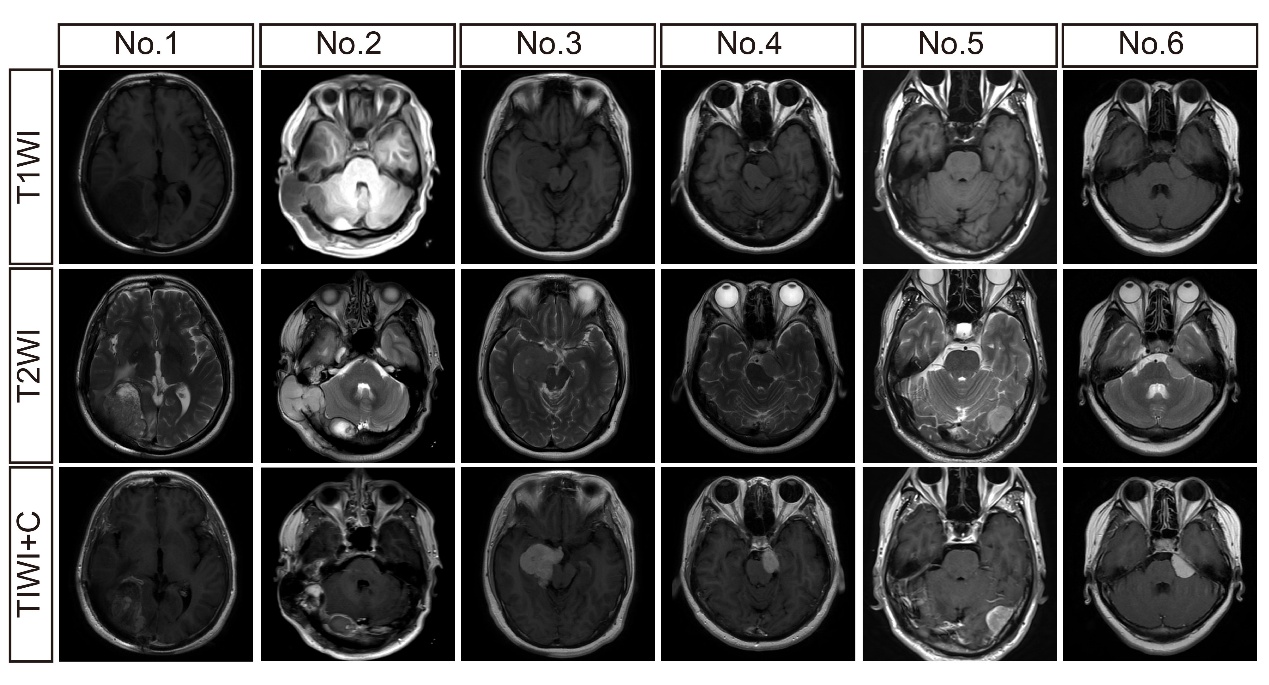


Additional file 11: Figure S11. MRI images of T1-weighted (T1WI), T2-weighted (T2WI), and T1-weighted contrast-enhanced (T1WI+C) sequences from 6 patients with organoids (No.1–6)


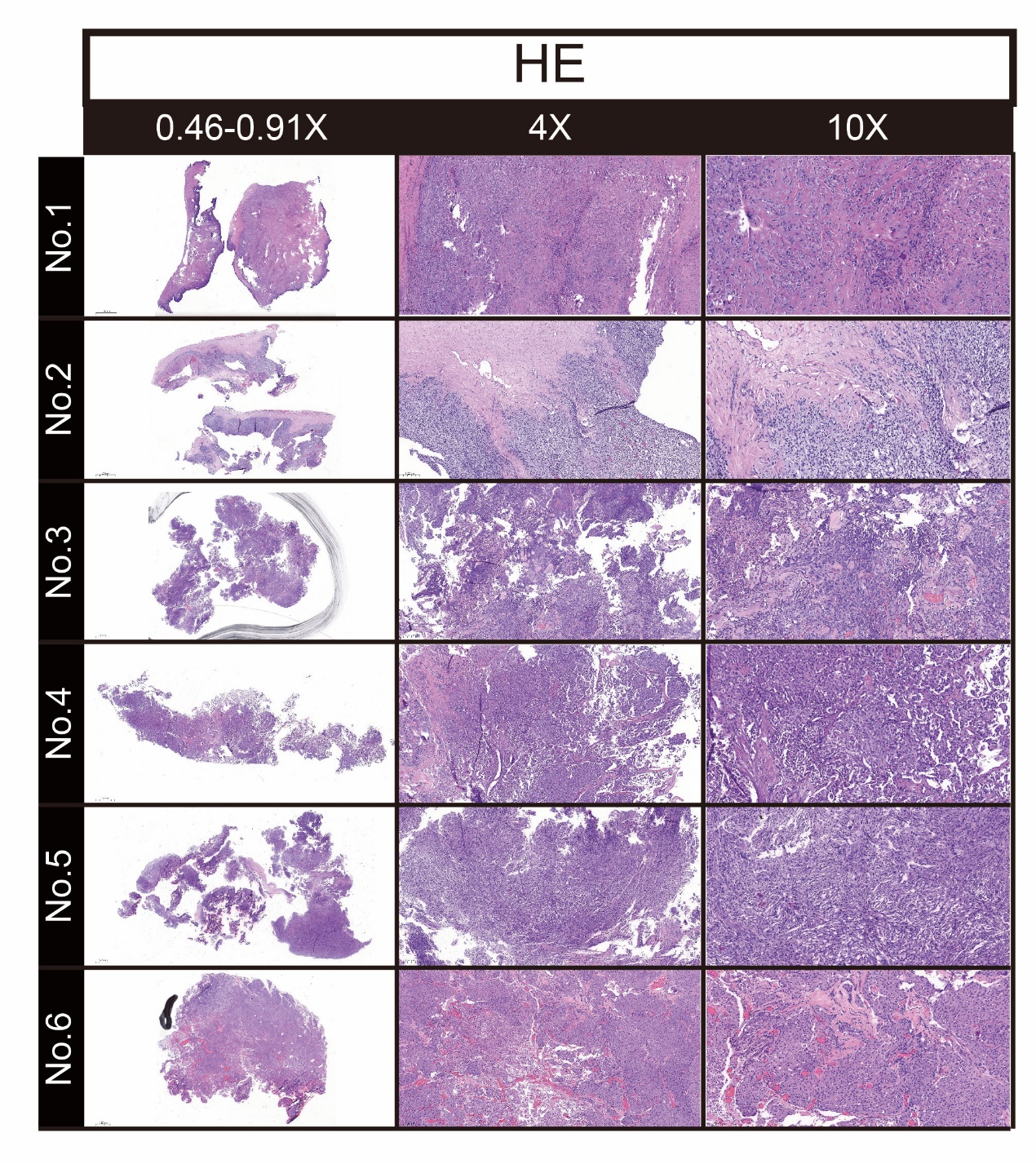


Additional file 12: Figure S12. Low-magnification field-of-view images at three different magnifications (0.46-0.91X, 4X, and 10X) to meningioma patients (No.1–6)


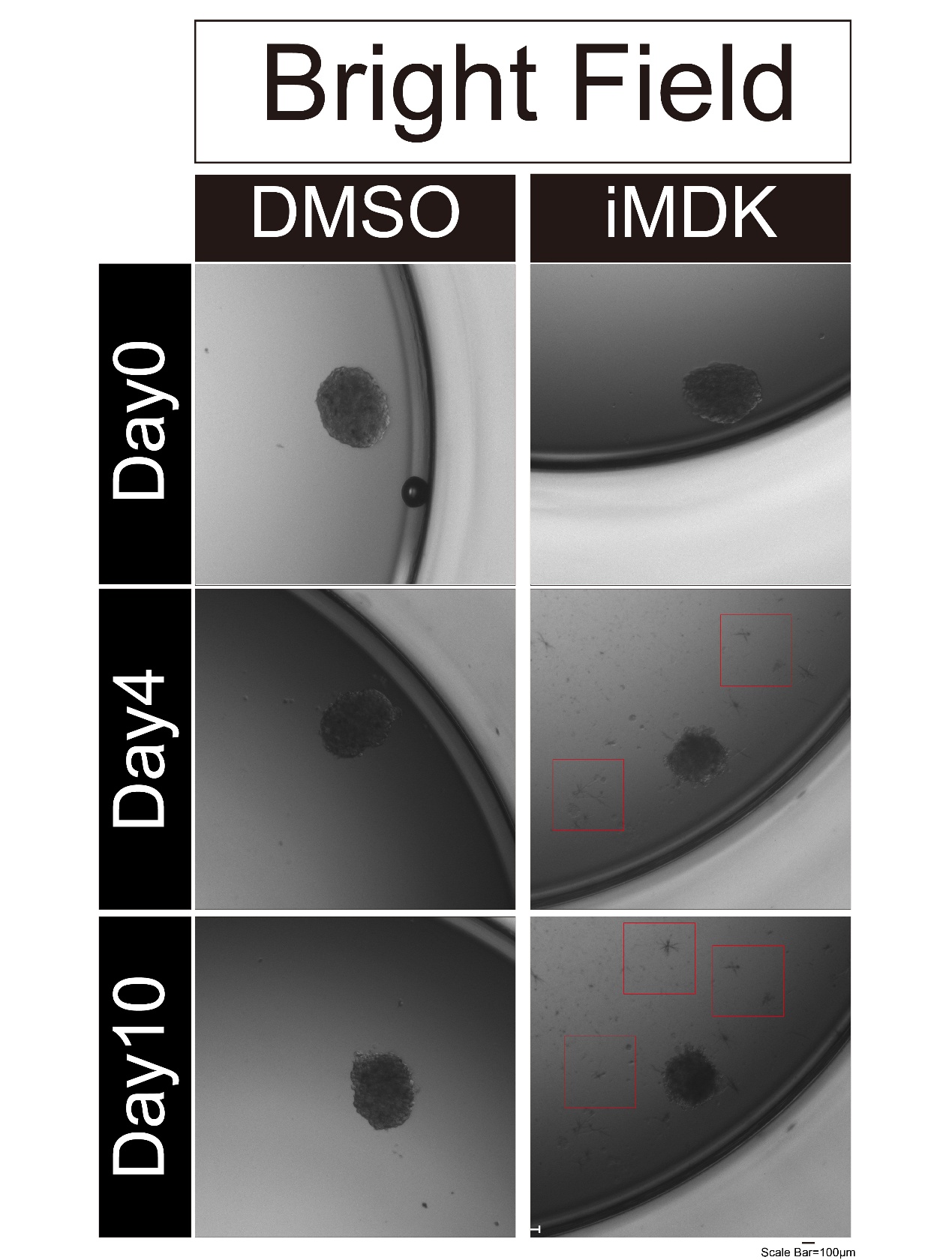


Additional file 13: Figure S13. Bright-field observation of tumor organoids in DMSO control (left column) and iMDK-treated (right column) groups, showing morphological changes at Day 0, Day 4, and Day 10 of culture; red boxes indicate the features of peripheral cell shedding and irregular contours after iMDK treatment.

Additional file 14: Table S1. Clinical information of patients whose samples were used for WB.

| Characteristics | WB |
| --- | --- |
| Patient number | 21 |
| Age,mean(SD),yr  PFS,mean(SD),mon  Sex  Male  Female | 46.6(15.4)  50.7(28.3)  10(47.6%)  11(52.4%) |
| WHO Grade |  |
| 1 | 15(71.4%) |
| 2  3  Recurrence  Y  N | 5(23.8%)  1(4.8%)  10(47.6%)  11(52.4%) |

Additional file 15: Table S2. Clinical information of patients whose samples were used for ELISA.

| Characteristics | ELISA |
| --- | --- |
| Patient number | 20 |
| Age,mean(SD),yr  Sex  Male  Female | 53.8(12.5)  9(45.0%)  11(55.0%) |
| WHO Grade |  |
| 1 | 19(95.0%) |
| 2  Adverse pathological  Y  N | 1(5.0%)  8(40.0%)  12(60.0%) |

Additional file 16: Table S3. Clinical information of patients whose samples were used for IHC staining.

| Characteristics | IHC |
| --- | --- |
| Patient number | 48 |
| Age,mean(SD),yr  PFS,mean(SD),mon  Sex  Male  Female | 45.8(10.8)  49.3(31.4)  23(47.9%)  25(52.1%) |
| WHO Grade |  |
| 1 | 45(93.8%) |
| 2  3  Recurrence  Y  N | 2(4.2%)  1(2.1%)  24(50.0%)  24(50.0%) |

Additional file 17: Table S4. Clinical information of patients whose samples were used for multiplex immunofluorescence staining.

| Characteristics | mIF |
| --- | --- |
| Patient number | 24 |
| Age,mean(SD),yr  Sex  Male  Female | 47.8(12.6)  11(45.8%)  13(54.2%) |
| WHO Grade |  |
| 1 | 22(91.7%) |
| 2  MDK expreaaion  High  Low | 2(8.3%)  12(50.0%)  12(50.0%) |

Additional file 18: Table S5. Clinical information of patients whose samples were used for meningioma organoids.

| No. | Age,yr | Sex | WHO Grade | Recurrence | Radiotherapy | Ki67 |
| --- | --- | --- | --- | --- | --- | --- |
| No.1 | 64 | M | 2 | Y | N | 10-15% |
| No.2 | 37 | M | 3 | Y | N | 20-30% |
| No.3 | 33 | F | 3 | Y | Y | 10-20% |
| No.4 | 56 | F | 1 | N | N | 2-8% |
| No.5 | 65 | M | 1 | N | N | 3-6% |
| No.6 | 48 | F | 1 | N | N | 2-5% |

Additional file 19: Table S6. Primer sequences.

| Primer name | primer sequences (5'to3') |
| --- | --- |
| MDK forward | CGCGGTCGCCAAAAAGAAAG |
| MDK reverse | TACTTGCAGTCGGCTCCAAAC |
| GAPDH forward | GGAGCGAGATCCCTCCAAAAT |
| GAPDH reverse | GGCTGTTGTCATACTTCTCATGG |

Additional file 20: Table S7. Antibody Information.

| Target | Dilution | Application | Manufacturer | Catalog# |
| --- | --- | --- | --- | --- |
| MDK- | 1:1000 | WB | Abcam | ab52637 |
| MDK | 1:50 | IHC-P | Abcam | ab52637 |
| SSTR-2 | 1:800 | IHC-P | Proteintech | 20404-1-AP |
| β-Actin | 1:2000 | WB | CST | 4967S |
| Vimentin  CD4  CD8 | 1:400  1:500  1:2000 | IHC-P  IHC-P  IHC-P | CST  Abcam  Abcam | 5741S  133616  237709 |
| HRP-conjugated Goat Anti-Rabbit IgG(H+L) | 1:20000 | WB | Proteintech | SA00001-2 |
| Goat Anti-Mouse IgG, HRP Conjugated | 1:20000 | WB | cwbio | CW0102S |

Additional file 21: Table S8. Differentially expressed genes in recurrence-related subclusters based on the internal dataset.

Additional file 22: Table S9. Differentially expressed genes in recurrence-related subclusters based on the external dataset.

Additional file 23: Table S10. The number of each state of CD8⁺ T cells in Scissor1 and Scissor2, based on internal dataset.

Additional file 24: Table S11. The number of each state of CD4⁺ T cells in Scissor1 and Scissor2, based on internal dataset.

Additional file 25: Table S12. The number of each state of CD8⁺ T cells in Scissor1 and Scissor2, based on external dataset.

Additional file 26: Table S13. The number of each state of CD4⁺ T cells in Scissor1 and Scissor2, based on external dataset.
